# Association between ultra-processed foods and risk of cancer: a systematic review and meta-analysis

**DOI:** 10.3389/fnut.2023.1175994

**Published:** 2023-06-08

**Authors:** Ying Lian, Gang-Pu Wang, Guo-Qiang Chen, Hua-Nan Chen, Guang-Yong Zhang

**Affiliations:** ^1^Department of Health Management and Engineering Laboratory for Health Management, The First Affiliated Hospital of Shandong First Medical University and Shandong Provincial Qianfoshan Hospital, Jinan, China; ^2^Department of Medical Record Management and Statistics, Shandong Provincial Qianfoshan Hospital and The First Affiliated Hospital of Shandong First Medical University, Jinan, China; ^3^Department of General Surgery, The Fourth People's Hospital of Jinan City, Jinan, China; ^4^Department of General Surgery, Shandong Provincial Qianfoshan Hospital and The First Affiliated Hospital of Shandong First Medical University, Jinan, China

**Keywords:** ultra-processed foods (UPFs), colorectal cancer, breast cancer, systematic review, meta-analysis

## Abstract

**Background:**

Despite increasing evidence that has shown the association of ultra-processed foods (UPFs) with cancer risk, the results remain inconclusive. We, therefore, conducted the meta-analysis to clarify the association by including recently published studies.

**Methods:**

A comprehensive search was conducted in PubMed, Embase, and Web of Science to identify all relevant studies from inception to January 2023. To pool data, fixed-effects or random-effects models were used where appropriate. Subgroup analyses, sensitivity analyses, and publication bias tests were performed.

**Results:**

A total of 13 studies (4 cohort studies and 9 case–control studies) were included in the analysis, with a total of 625,738 participants. The highest UPFs consumption was associated with increased risk of colorectal cancer (OR = 1.23, 95% CI: 1.10–1.38), colon cancer (OR = 1.25, 95% CI: 1.14–1.36), and breast cancer (OR = 1.10, 95% CI: 1.00–1.20) but not rectal cancer (OR = 1.18, 95% CI: 0.97–1.43) and prostate cancer (OR = 1.03, 95% CI: 0.93–1.12). In addition, the subgroup analyses showed that a positive association between UPFs consumption and colorectal cancer was observed among men (OR = 1.31, 95% CI: 1.15–1.50), whereas no significant association was observed among women (OR = 1.10, 95% CI: 0.94–1.29).

**Conclusion:**

The present meta-analysis suggests that high UPFs consumption is associated with a significantly increased risk of certain site-specific cancers, especially the digestive tract and some hormone-related cancers. However, further rigorously designed prospective and experimental studies are needed to better understand causal pathways.

## 1. Introduction

Cancer is one of the leading causes of death worldwide (1). According to a report from the World Health Organization, cancer is responsible for almost 10 million deaths per year, and every sixth death in the world is attributed to cancer (2, 3). It is expected that new cases of cancer will increase to 28.4 million by 2040, and the burden of cancer will double in the next 20 years. Therefore, there is a need for more research on exploring and intervening in potential risk factors for cancer. It is reported that a substantial proportion of cancer cases could be prevented by eliminating risk factors (4). In addition to genetic predisposition, numerous modifiable factors have also been implicated in regulating tumorigenesis and cancer development, such as a sedentary lifestyle (5) and unhealthy dietary patterns (6). Thus, further study on lifestyle modification is warranted to better identify targets for the intervention of cancer.

Evidence of the link between the degree of food processing and increased cancer risk is emerging (7). Recent global estimates demonstrate dramatical changes in the processing of foodstuffs, which have witnessed a marked increase in processed food availability, especially during the historically unprecedented SARS-CoV-2 pandemic lockdown setting (8), with ultra-processed foods (UPFs) accounting for more than half of total energy intake (9). Indeed, UPFs are usually characterized by their poor nutritional composition, high energy density, and the presence of components derived from food processing or packaging, with potential carcinogenic properties. Previous studies have investigated the possible linkage between UPFs consumption and chronic non-communicable diseases (10, 11) and related morbidity (12) and mortality (13), including three systematic reviews on cancer (14–16). Nevertheless, existing systematic reviews evaluating the associations of UPFs consumption with cancer did not get quantitative synthesis results limited by the number of studies available for inclusion (14). In addition, although there is evidence suggesting the potential carcinogenic pathways underlying the association between UPFs and cancer risk, previous studies have only focused on the most common cancer sites, such as breast cancer, and no previous study has assessed the effect of UPFs on a comprehensive range of cancers. Furthermore, several additional studies have been published on the effect of UPFs consumption on various types of cancer (17); however, these results are conflicting, leading to insufficient generalizability of the findings.

To bridge the knowledge gap, in the present study, we conducted the current comprehensive and updated systematic review and further explored the association between UPFs consumption and different types of cancer.

## 2. Methods

The present systematic review and meta-analysis were carried out in accordance with the 2020 Preferred Reporting Items for Systematic Reviews and Meta-Analyses (PRISMA) guidelines (18).

### 2.1. Search strategy

The electronic databases of PubMed, Embase, and Web of Science were comprehensively searched for relevant studies from inception to January 2023. The following search terms were used: (ultra-processed OR processed food OR ultraprocessed) and (neoplasm OR tumor^*^ OR cancer^*^ OR malignant^*^ OR carcinoma OR adenocarcinoma OR neoplasia). There were no restrictions on language. Further studies and relevant gray literature were manually searched by checking the references of the potentially eligible articles.

### 2.2. Inclusion and exclusion criteria

This review included observational studies (cross-sectional, cohort, and case–control) that investigated the association between UPFs consumption and cancer risk and reported the results as relative risks (RRs) or odds ratios (ORs) with 95% confidence intervals (CIs). The UPFs were defined by the NOVA food classification system. The outcome of interest is specific cancer type, and non-malignant abnormalities (e.g., adenomas) were not considered. We excluded experimental studies, review articles, letters, editorials, and abstracts without full texts.

### 2.3. Data extraction and quality assessment

Data extraction was carried out from eligible articles using a predefined checklist. The following information was extracted: the first author's name, year of publication, country, design, follow-up time (for cohort studies), total subjects, the number of cases, type of cancer, age, gender, exposure, methods of exposure assessment, ORs, or RRs (95% CIs), and adjusted (confounding) variables. The Newcastle–Ottawa Quality Assessment Scale (NOS) was used to assess the quality of the included studies (19). Scores ranged from 0 to 9 with a score of ≥7 being considered as of high quality. Data collection and quality assessment processes were independently performed by YL and G-PW. Any discrepancies in data extraction and quality assessment were resolved by discussion with the third author.

### 2.4. Statistical analysis

All data were analyzed using STATA version 14.0 (StataCorp, College Station, TX, USA). The ORs with 95%CIs for UPF consumption and cancer risk were pooled using fixed-effects or random-effects models where appropriate. Heterogeneity was assessed using the *I*^2^ value and Q-test (P-heterogeneity). If the P-heterogeneity of the Q-test ≤0.10 or *I*^2^ ≥50% indicated a high degree of heterogeneity among studies, then a random-effects model was used. UPFs consumption was analyzed as a continuous variable (per 10% increment) and as a categorical variable. Subgroup analyses were conducted according to a series of key variables that might influence the association between UPFs and cancer, including tumor subtype, sex (for colorectal), and menopausal status (for breast cancer). Sensitivity analyses were carried out by removing each study and recalculating the pooled effect estimates (i.e., one study removed analysis). Publication bias was assessed by formal testing by Egger's test and Begg's test.

## 3. Results

### 3.1. Study characteristics

**Figure 1:**
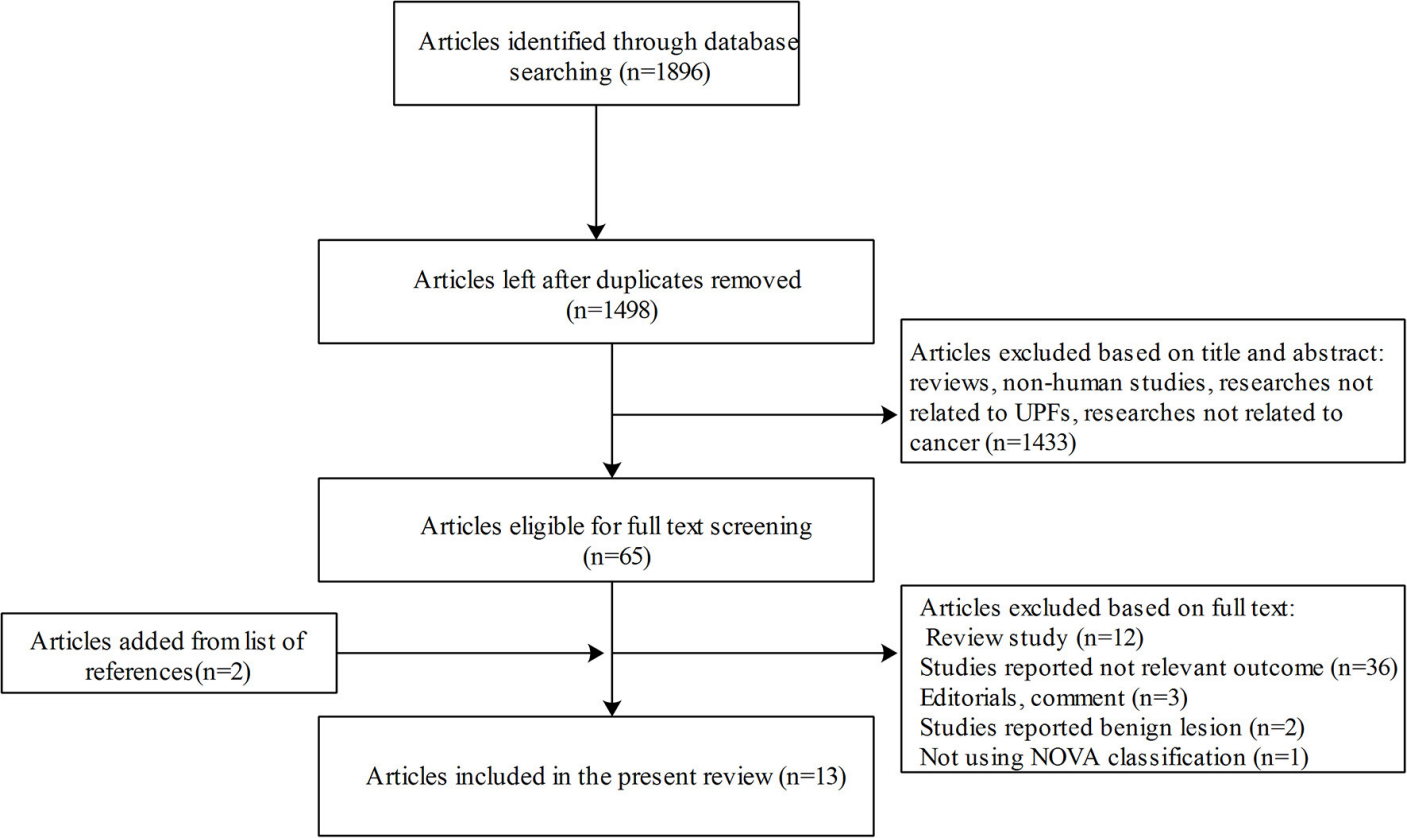
Flow diagram of study selection.

The flow chart of the literature screening and selection process is presented in Figure 1. A total of 13 studies met our inclusion criteria and were included in the present systemic review (17, 20–31). All the studies with a total sample size of 625,738 participants were published from 2018. Of these studies, four were cohort studies and nine were case–control designs. In total, five studies were conducted in America, five in Europe, two in Africa, and one in Asia. Of the 13 eligible studies, six focused on colorectal cancer, six on breast cancer, four on prostate cancer, and two on pancreatic cancer, chronic lymphocytic leukemia, and central nervous system tumors. The degree of processing of foods was classified according to the NOVA classification system. The general characteristics of included studies are described in Table 1.

**Table 1:** Characteristics of included studies.

**Author**	**Location**	**Sample size**	**Sex: female, %**	**Age**	**Design of study**	**Type of cancer**	**UPFs assessment**	**Comparison**	**NOS score**
Romaguera et al. (20)	Spanish	7,834	49.9	62.9 ± 11.9	CCS	Colorectal, breast, and prostate cancer	FFQ	Tertile 3 vs. Tertile 1. Each 10% increase of UPFs	7
Fiolet et al. (21)	French	104,980	78.26	42.8 ± 14.8	CS	Colorectal, breast, and prostate cancer	24 h dietary records	Quartile4 vs. Quartile 1. Each 10% increase of UPFs	8
Jafari et al. (29)	Iran	213	NR	40–75	CCS	Colorectal cancer	FFQ	Tertile 3 vs. Tertile 1	8
Wang et al. (17)	America	206,248	77.53	25–75	CS	Colorectal cancer	FFQ	Quintile 5 vs. Quintile 1. Each 10% increase of UPFs	8
Trudeau et al. (22)	Canada	3,910	0	64 ± 7	CCS	Prostate cancer	FFQ	Quartile4 vs. Quartile 1	7
Jacobs et al. (23)	African	792	100	54.6 ± 12.9	CCS	Breast cancer	QFFQ	Tertile 3 vs. Tertile 1	8
El Kinany et al. (24)	Morocco	2,906	50.7	56.0 ± 13.9	CCS	Colorectal cancer	FFQ	Tertile 3 vs. Tertile 1	8
Solans et al. (25)	Spanish	1,864	41.4	63.9 ± 10.8	CCS	Chronic lymphocytic leukemia	FFQ	Tertile 3 vs. Tertile 1. Each 10% increase of UPFs	7
Zhong et al. (26)	America	98,265	52.53	65.6 ± 5.7	CS	Pancreatic cancer	DHQ	Quartile4 vs. Quartile 1	8
Romieu et al. (27)	Latin American	1,050	100	40 (31–45)	CCS	Breast cancer	FFQ	Tertile 3 vs. Tertile 1	8
Queiroz et al. (30)	Brazil	118	100	53.1 ± 13.8	CCS	Breast cancer	FFQ	Categories of UPFs	8
Chang et al. (31)	UK	197426	54.6	58.0 ± 8.0	CS	Various site-specific cancers	24 h dietary records	Quintile 5 vs. Quintile 1. Each 10% increase of UPFs	8
Esposito et al. (28)	Italy	132	40.9	54.3 ± 13.5	CCS	Central nervous system tumors	FFQ	Quartile4 vs. Quartile 1	8

UPFs, ultra-processed foods; NR, no report; FFQ, food frequency questionnaire; DHQ, a diet history questionnaire; CCS, case–control study; CS, cohort study; NOS, Newcastle–Ottawa Quality Assessment Scale.

### 3.2. Meta-analysis

#### 3.2.1. UPFs consumption and colorectal cancer risk

**Figure 2:**
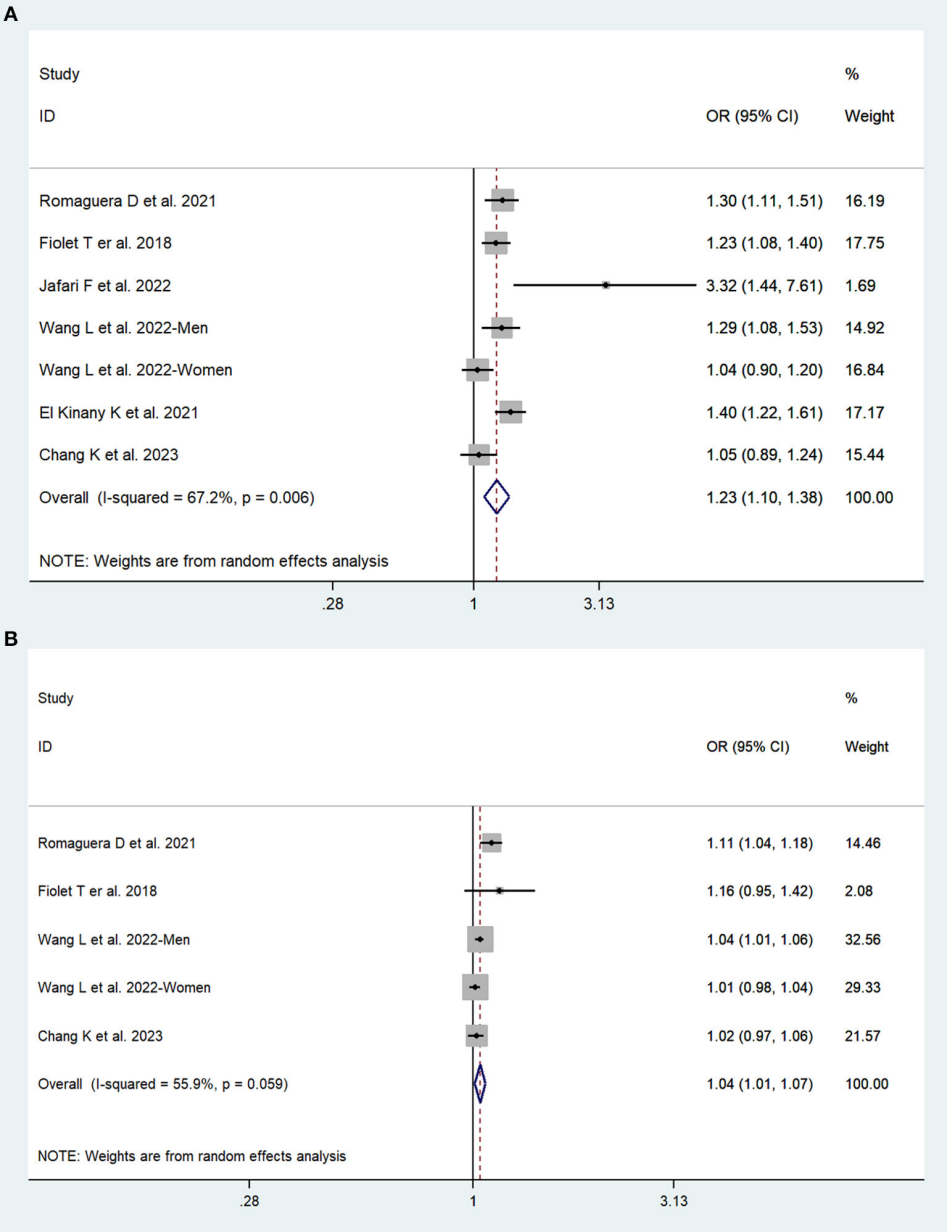
Forest plots of pooled ORs for UPFs and colorectal cancer. **(A)** The highest category UPFs compared with the lowest category UPFs. **(B)** 10% increase in UPFs consumption.

In total, three prospective cohort studies with a total of 508,654 participants and three case–control studies with a total of 8,424 participants reported the association between UPFs consumption and the risk of colorectal cancer. The highest consumption of UPFs was found to be associated with an increased risk of colorectal cancer. The pooled OR was 1.23 (95% CI: 1.10–1.38), with moderate evidence of heterogeneity (*I*^2^ = 67.2%, *P* = 0.01, Figure 2A). There was no evidence of significant publication bias with Begg's test (*P* = 0.54) and Egger's test (*P* = 0.27). Sensitivity analyses suggested that the pooled estimate of colorectal cancer risk did not apparently modify any one study, confirming the stability of the present results. Each 10% increase in UPFs consumption was associated with a 4% higher risk of colorectal cancer (OR = 1.04, 95% CI: 1.01–1.07; *I*^2^= 55.9%, *P*=0.06, Figure 2B). Subgroup analyses showed that a positive association between UPF consumption and colorectal cancer was observed among men (OR = 1.31, 95% CI: 1.15–1.50), whereas no significant association was observed among women (OR = 1.10, 95% CI: 0.94–1.29). The subgroup analyses are presented in Table 2.

**Table 2:** Subgroup analyses for UPFs consumption and cancer risk.

**Type of cancer**	**Number of studies**	**OR (95% CI)**	***I^2^*(%)**	***P***
**Colorectal cancer**	6			
Categorical variable	6	1.23 (1.10–1.38)	67.2	0.01
Continuous variable 10% increase in UPFs	4	1.04 (1.01–1.07)	55.9	0.06
Anatomic subsites				
Colon cancer	4	1.25 (1.14–1.36)	19.3	0.28
Rectal cancer	4	1.18 (0.97–1.43)	62.0	0.03
Gender				
Men	2	1.31 (1.15–1.50)	0	0.78
Women	2	1.10 (0.94–1.29)	29.0	0.24
**Breast cancer**	6			
Categorical variable	6	1.10 (1.00–1.20)	45.4	0.10
Continuous variable 10% increase in UPFs	3	1.03 (0.98–1.09)	58.8	0.09
Menopausal status				
Premenopausal breast cancer	5	1.24 (0.95–1.60)	50.2	0.09
Postmenopausal breast cancer	4	1.08 (0.96–1.20)	40.2	0.17
**Prostate cancer**	4			
Categorical variable	4	1.03 (0.93–1.12)	0	0.75
Continuous variable 10% increase in UPFs	3	0.99 (0.96–1.02)	0	0.83
Tumor aggressiveness				
Low-grade prostate cancers	2	0.93 (0.76–1.14)	0	0.60
High-grade prostate cancers	2	1.05 (0.84–1.32)	0	0.56
**Pancreatic cancer**	2	1.24 (0.98–1.57)	59.8	0.12
**Chronic lymphocytic leukemia**	2	1.08 (0.80–1.44)	0	0.93
**Central nervous system tumors**	2	1.20 (0.87–1.66)	69.2	0.07

#### 3.2.2. UPFs consumption and breast cancer risk

**Figure 3:**
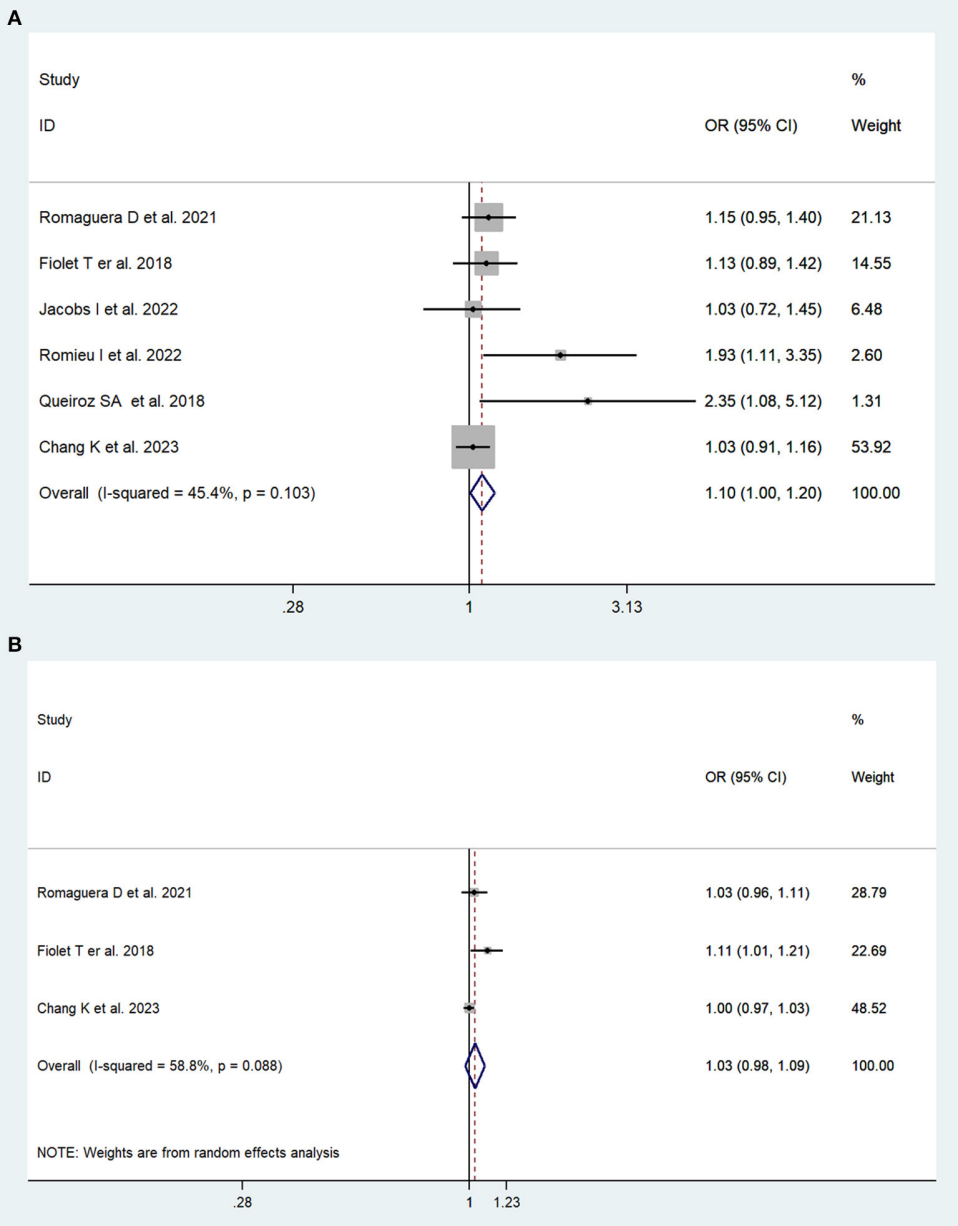
Forest plots of pooled ORs for UPFs and breast cancer. **(A)** The highest category UPFs compared with the lowest category UPFs. **(B)** 10% increase in UPFs consumption.

In total, two cohort studies with a total of 279,585 participants and four case–control studies with a total of 5,059 participants assessed the link between UPFs consumption and breast cancer risk. This meta-analysis showed that greater UPFs consumption was associated with higher odds of breast cancer (OR: 1.10, 95%CI: 1.00–1.20). Heterogeneity between studies was not significant (*I*^2^ = 45.4%, *P* = 0.10, Figure 3A). Publication bias was tested by Egger's test (*P* = 0.03) and Begg's test (*P* = 0.19). Each 10% increase in UPFs consumption was not associated with the risk of breast cancer (OR = 1.03, 95% CI: 0.98–1.09, *I*^2^= 58.8%, *P* = 0.09, Figure 3B).

#### 3.2.3. UPFs consumption and prostate cancer risk

**Figure 4:**
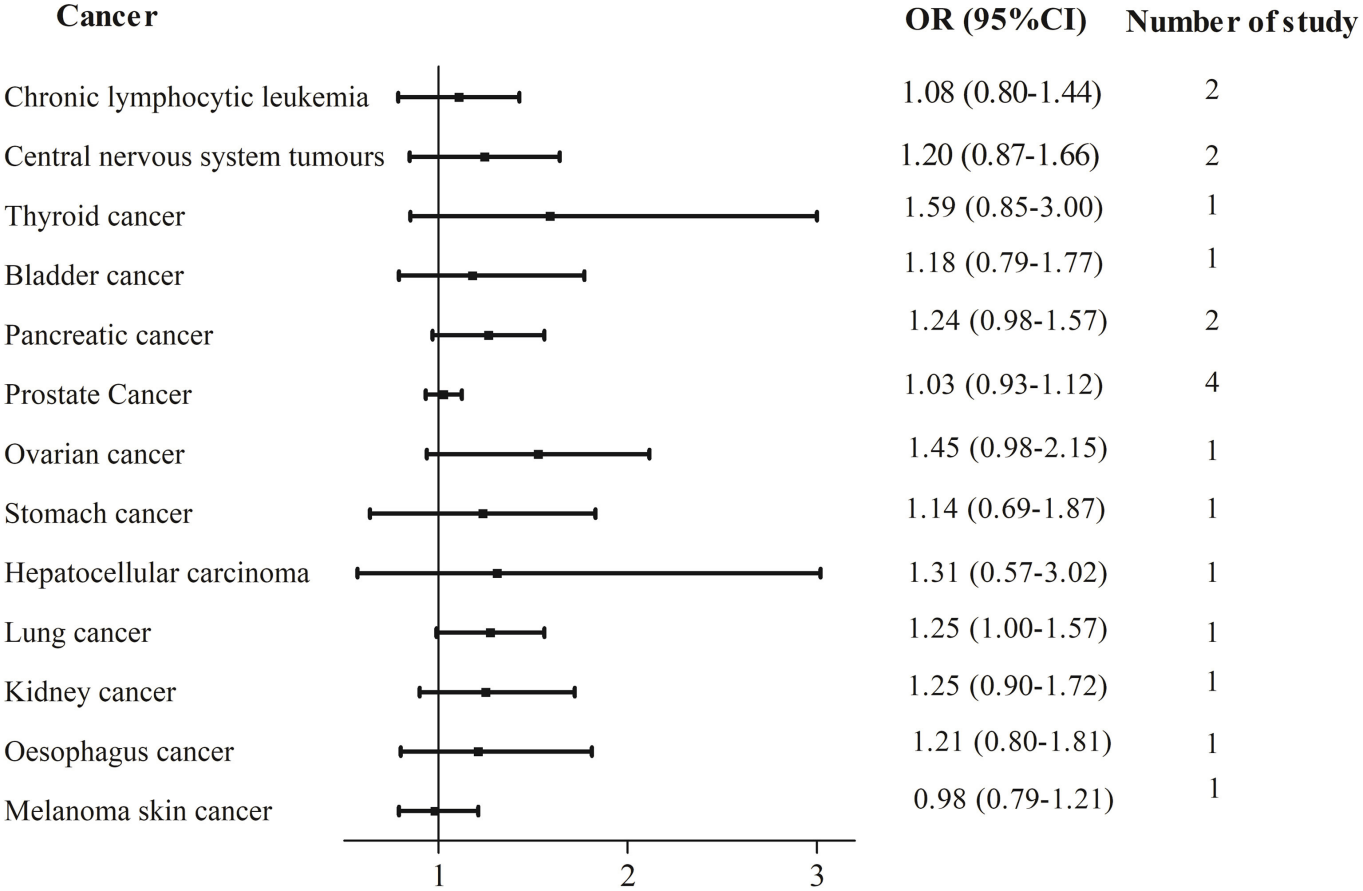
Forest plots of pooled ORs for UPFs and other types of cancer.

In total, two cohort studies with a total of 220,247 participants and two case–control studies with a total of 6,123 participants reported the association between UPFs consumption and prostate cancer risk. There was no significant association between UPFs consumption and prostate cancer. The pooled OR (95%CI) for the highest UPFs consumption was 1.03 (0.93–1.12), with no significant heterogeneity between studies (*I*^2^ = 0%, *P* = 0.75, Figure 4).

#### 3.2.4. UPFs consumption and other types of cancer

In total, two studies were available regarding three other types of cancer including pancreatic cancer, chronic lymphocytic leukemia, and central nervous system tumors. One study investigated the link between UPFs consumption and other multiple cancer sites with the findings shown in Figure 4.

## 4. Discussion

The present systematic review comprehensively quantified the association between UPFs consumption and various types of cancer risk integrating four prospective cohort studies and nine case–control studies. Our findings indicated that greater intake of UPFs was associated with increased odds of colorectal and breast cancer. Every 10% increase in the proportion of UPFs in the diet was associated with a 4% higher risk of colorectal cancer. In addition, the results of subgroup analyses proposed that a significant association of UPFs consumption with an increased risk of colorectal cancer was noted in men but not among women.

Our findings provided robust evidence that a high intake of processed foods increases the risk of colorectal cancer, which has been previously reported in recent systematic reviews (32). For example, a meta-analysis of prospective cohort studies showed that compared with the lowest category of processed meat intake, the highest category was associated with higher overall colorectal cancer risk (33). Similarly, significant positive associations were also observed for colon cancer. In addition, our results are also consistent with previous meta-analyses and broaden whole of dietary pattern analyses. The systematic reviews found that the dietary inflammatory index characterized by excess consumption of processed foods, including processed meats, sweets, fried foods, and refined grains appears to be associated with cancer risk (34), while Mediterranean-style diets, which are rich in fruits, vegetables, extra virgin olive oil, whole grains, and other unprocessed or minimally processed foods, reduce the risk of colorectal cancer by 17% (35). However, these dietary patterns are often unable to determine the industrial processing level of foods. The objective and standardized criteria of the NOVA classification system were used in all the included studies to distinguish UPFs from other foods based on the nature, extent, and purpose of food processing (36–38), which can provide novel insights into understanding the role of food processing level in the development of cancer (39). Of note, the stratified analyses showed a positive association between UPFs consumption and increased risk of colorectal cancer in men but not among women. The findings are somewhat concordant with another previous ultra-processed food inflammation study, which suggests that men are more predisposed to the carcinogenic effects of diet (40). Potential explanations for such different sex patterns may involve the effect of sex hormones or genetics (41). Further studies are required to clarify these findings.

In the analysis of breast cancer, a positive association was found between higher UPFs consumption and breast cancer risk, which is consistent with those from the prior meta-analyses. Previously, a meta-analysis combining data from 15 studies showed that the highest processed meat intake was related to a 9% increased risk of breast cancer compared with the lowest intake (32). In another previous analysis, a similar magnitude positive association was found between processed meat intake and breast cancer risk by comparing the highest with the lowest category (42). It seems that menopausal status may influence the association between UPFs consumption and breast cancer risk. It was found that higher processed meat consumption was associated with a 9% greater risk of postmenopausal breast cancer; however, such a positive association was not observed for premenopausal breast cancer (42). The present study examining the association by menopausal status suggested no significant associations with the intake of UPFs for breast cancer in premenopausal and postmenopausal women. In addition, breast cancer is a heterogeneous disease, with potentially distinct etiology for different hormone receptor statuses, and it has been suggested that estrogen receptor-positive breast tumors (ER+) are more strongly associated with hormone-related factors than estrogen receptor-negative tumors (ER-) (43); therefore, assessing risk factors for breast cancer incorporating molecular pathological information may confer even greater insights (44). ER status was reported in one included study; it shows a significant association for UPFs among ER + breast cancer, while no association was observed in ER-tumor risk (27). Thus, further studies are required to understand the heterogeneity of this relationship by molecular subtypes according to the menopausal status of breast cancer.

The present meta-analysis has some strengths. This is the first meta-analysis comprehensively quantitatively summarizing the evidence on the association between UPFs consumption and various types of cancer, providing strong implications for dietary policies and guidelines. In addition, the present meta-analysis included large sample size and high-quality epidemiological data, with the standardized assessment of processed diet intake using the NOVA system, along with sensitivity analyses and detailed subgroup analyses, ensuring greater precision and reliability of the results. Despite the interesting results of the present meta-analysis, some limitations should be considered. First, although we include several prospective cohorts with large sample sizes, some of the included studies are case–control designs, which does not allow for the identification of a causal link between the exposure and outcome. Second, cancer is often described as the result of complex interactions between biological, social, and psychological factors, although most included studies have adjusted for a wide range of potential confounders, other unmeasured or inadequately measured factors, for example, genetic and environmental factors, may result in residual confounding. Third, of the articles included, UPFs intake was generally evaluated through food frequency questionnaires or food records that were not specifically designed to identify UPFs, which can result in some degree of misclassification error, thus leading to bias associations. Further well-designed studies that address such limitations are warranted to confirm the associations.

Although the underlying pathways of our findings have not yet been fully elucidated, several mechanisms have been proposed to account for the potential carcinogenicity of UPFs. First, UPFs often have a poorer nutritional quality compared to minimally processed foods, which tend to be rich in unfavorable nutritional components, such as saturated fat, added sugar, energy density, and salt, along with lower fiber and vitamins. Meanwhile, a randomized controlled trial conducted in inpatients found that more ultra-processed diet intake could lead to excess calorie intake and substantial weight gain (45). Poor diet quality together with obesogenic properties are all important factors in driving their detrimental impact on cancer. Second, food additives in the processing or packaging of UPFs, such as emulsifiers, preservatives, colors, and flavors, have also been suggested as potential mechanisms linking UPFs to higher cancer risk. Some contaminants in UPFs have been linked to proinflammation potential (46), endocrine-disrupting effects (47), and dysbiosis (48), which have been proven to promote carcinogenesis in epidemiological, clinical, and experimental studies. For example, it is notably suggested that consumption of UPFs was associated with an elevated level of inflammatory biomarkers, such as IL-6 concentration, which are involved in tumor progression at almost every step including initiation, progression, and metastasis (49). Moreover, consumption of UPFs may increase exposure to endocrine-disrupting chemicals, including bisphenol A and phthalates, leading to a persistent epigenetic change in genes and subsequently stimulating the proliferation of hormone-sensitive tissues in a tumor sense. In addition, UPFs could also alter gut microbiota composition and function unfavorably (50), which, in turn, increase cancer risk through multiple molecular signals, including inhibiting T-cell activity and promoting DNA damage (51). Further investigation into the mechanistic pathways is warranted to better identify targets for intervention.

## 5. Conclusion

The present systematic review showed that the high consumption of UPFs was associated with an increased risk of certain site-specific cancers, especially the digestive tract and some hormone-related cancers including colorectal and breast, providing a more comprehensive understanding of the potential implications in the development of cancer associated with processed diet. The findings support the importance of public health by boosting prevention policies to limit UPFs consumption and promoting healthier nutritional status for primary cancer prevention. Furthermore, well-designed studies are needed to better strengthen the evidence of the association between UPFs and cancer risk.

## Author contributions

G-YZ conceived and designed the study. G-PW, H-NC, G-QC, and YL analyzed the data. G-PW and YL wrote the manuscript. All authors provided critical revisions of the manuscript and approved the final manuscript.
